# NGS-based targeted sequencing identified six novel variants in patients with Duchenne/Becker muscular dystrophy from southwestern China

**DOI:** 10.1186/s12920-023-01556-1

**Published:** 2023-05-30

**Authors:** Feng Tang, Yuanyuan Xiao, Cong Zhou, Haixia Zhang, Jing Wang, Yang Zeng

**Affiliations:** 1 Department of Medical Genetics, West China Second University Hospital of Sichuan University, Chengdu, People’s Republic of China; 2 Key Laboratory of Birth Defects and Related Diseases of Women and Children, Ministry of Education, Sichuan University, Chengdu, 610041 Sichuan People’s Republic of China

**Keywords:** DMD, BMD, Capture, NGS, Genetic counseling, Prenatal diagnosis

## Abstract

**Background:**

At present, Multiplex ligation-dependent probe amplification (MLPA) and exome sequencing are common gene detection methods in patients with Duchenne muscular dystrophy or Becker muscular dystrophy (DMD/BMD), but they can not cover the whole-genome sequence of the DMD gene. In this study, the whole genome capture of the DMD gene and next-generation sequencing (NGS) technology were used to detect the patients with DMD/BMD in Southwest China, to clarify the application value of this technology and further study the gene variant spectrum.

**Methods:**

From 2017 to 2020, 51 unrelated patients with DMD/BMD in southwestern China were clinically diagnosed at West China Second University Hospital of Sichuan University (Chengdu, China). The whole-genome of the DMD gene was captured from the peripheral blood of all patients, and next-generation sequencing was performed. Large copy number variants (CNVs) in the exon regions of the DMD gene were verified through MLPA, and small variations (such as single nucleotide variation and < 50 bp fragment insertions/deletions) were validated using Sanger sequencing.

**Results:**

Among the 51 patients, 49 (96.1% [49/51]) had pathogenic or likely pathogenic variants in the DMD gene. Among the 49 positive samples, 17 patients (34.7% [17/49]) had CNVs in the exon regions and 32 patients (65.3% [32/49]) had small variations. A total of six novel variants were identified: c.10916_10917del, c.1790T>A, c.1842del, c.5015del, c.5791_5792insCA, and exons 38–50 duplication.

**Conclusions:**

Pathogenic or likely pathogenic variants of the DMD gene were detected in 49 patients (96.1% [49/51]), of which 6 variants (12.2% [6/49]) had not been previously reported. This study confirmed the value of NGS-based targeted sequencing for the DMD gene expanding the spectrum of variants in DMD, which may provide effective genetic counseling and prenatal diagnosis for families.

## Introduction

The dystrophin (DMD) gene (MIM #300377), with a total length of 2.3 Mb, is located on chromosome Xp21. Variants in the DMD gene encoding the protein dystrophin lead to the X-linked allelic disorders Duchenne (DMD, MIM 310200) and Becker muscular dystrophy (BMD, MIM 300376) [1]. The DMD gene usually affects men and is carried by women [2]. DMD/BMD is the most frequently inherited muscle disease, affecting 1.5–28.2 out of 100,000 male newborns worldwide [3–7]. Children with DMD/BMD are usually asymptomatic at birth and during infancy. In childhood, patients begin to develop calf muscle pseudohypertrophy, and progressive skeletal muscle weakness, and they gradually lose the ability to walk [8]. Some children with DMD/BMD have varying degrees of cognitive impairment, patients with DMD usually die in their early twenties due to cardiopulmonary failure [9].

The DMD gene is the largest human gene found to date and consists of 79 exons and 78 introns [10]. Currently, there are no effective treatments for DMD/BMD. However, genetic diagnosis and counseling for probands can help reduce the transmission of pathogenic genes and prevent the birth of people with DMD/BMD. Clinical diagnosis of DMD/BMD is classified into phenotypic, pathological, and genetic diagnoses. The classic clinical phenotypes (such as significantly elevated serum creatine kinase (CK) and Creatine Kinase isoenzyme MB (CK-MB), calf muscle pseudohypertrophy, and electromyography injury) and the patient's family history can be used as the basis for clinical diagnosis. For patients with atypical symptoms, pathological diagnosis is necessary; however, surgery is considered a less acceptable option for younger patients. In addition, younger patients are in the early stages of disease progression; thus, pathological diagnosis is difficult. For prenatal diagnosis of DMD/BMD, genetic testing must be performed. At present, common methods for the genetic diagnosis of DMD/BMD include multiplex ligation-dependent probe amplification (MLPA) and exome sequencing (ES). The MLPA is used for the detection of large copy number variations (CNVs) in the 79 exons of DMD, but it is incapable of detecting minor variations (including single nucleotide variation and < 50 bp fragment insertion/deletion). With ES technology, all exons in the DMD gene and the surrounding flanking region, but not the deep-intronic region, can be detected.

To detect the whole gene region of DMD, whole-genome capture and next generation-sequencing (NGS) were used to detect regions such as 5′UTR, exons, introns, and 3′UTR. It can detect not only CNVs in the whole gene region of DMD but also minor variations. Compared to the MLPA or ES, whole-genome capture and NGS assays are more extensive, have larger sample sizes, and are relatively less costly. Consequently, in this study, the DMD gene capture and NGS were performed on 51 children with DMD/BMD in southwestern China to clarify the value of this technology in the application of DMD gene detection and to identify the gene variant spectrum. This study can provide important information for prenatal diagnosis and genetic counseling of patients with DMD/BMD.

## Materials and methods

### Patients

Fifty-one unrelated probands with DMD/BMD diagnosed by a clinical phenotype or a pathological biopsy were included in this study, and their parents were free of consanguineous marriages. All the patients underwent whole-genome capture of the DMD gene and NGS. Fifty-one unrelated families in southwestern China were examined, including 50 males and one female, with an average age of 6.4 ± 4.8 years and an average age of onset of 2.7 ± 2.3 years. Among the 51 children diagnosed with the disease, 47 (92.2% [47/51]) showed increased CK and CK-MB levels, and 42 (82.4% [42/51]) had a “movement disorder”. There were 21 patients (41.2% [21/51]) with gastrocnemius hypertrophy, six (11.8% [6/51]) with cardiac abnormalities, three (5.9% [3/51]) with cognitive disorders such as dysplasia, two (3.9% [2/51]) with rib abnormalities, and two (3.9% [2/51]) who died of lung failure. Forty-two patients (82.4% [42/51]) were admitted to the hospital for the first time due to a “movement disorder”, and nine patients (17.6% [9/51]) due to a “markedly elevated CK/ CK-MB level”. We provide an overview of personal information, age at first onset/diagnosis, clinical phenotype, muscle biopsy results, and family history (Table 1).

**Table 1:** Basic clinical information of 51 Patients

No	Sex	Age (years)		Phenotype	Clinical manifestations								Positive muscle biopsy	Family history
		At onset	At diagnosis		Movement disorders	Wheelchair dependency	Elevated CK/ CK-MB level	Gastrocnemius hypertrophy	Cardiac abnormalities	Lungs failure	Rib abnormalities	Cognitive disorders		
1	M	2	11	DMD	+	+	+	+				+		+
2	M	1	10	DMD	+	+	+							
3	M	6	7	MD	+		+			+				+
4	M	1	6	DMD	+		+						+	
5	M	3	8	MD	+		+							+
6	M	2	6	MD	+		+	+						
7	M	5	19	DMD	+	+								
8	M	2	2	MD	+		+							
9	M	1	9	MD			+	+					+	
10	M	2	10	MD	+		+							+
11	M	7	8	MD	+		+				+			
12	M	2	3	MD	+		+							
13	M	0.16	2	MD	+		+							
14	M	3	6	MD	+		+	+						
15	M	3	3	MD	+		+							
16	M	4	5	MD			+	+						
17	M	3	4	MD	+		+							+
18	M	2	6	MD	+		+	+						
19	M	4	8	MD	+		+							
20	M	5	10	MD	+		+	+					+	+
21	M	0.66	0.66	MD			+							
22	M	1	6	MD	+		+	+					+	
23	M	3	8	MD	+		+							
24	M	1	1	MD	+		+							
25	M	2	2	MD			+	+				+		
26	M	2	2	MD	+		+	+						
27	M	2	20	DMD	+	+	+	+		+				
28	M	3	3	MD	+		+							
29	M	3	4	MD	+		+					+		
30	M	3	6	MD	+		+							+
31	M	1	7	MD	+		+							
32	M	1	2	MD	+		+	+	+					
33	F	6	6	MD			+		+					
34	M	5	6	MD	+		+	+						
35	M	0.33	1	MD			+							
36	M	3	6	MD	+		+	+	+					
37	M	1	3	MD	+		+	+						
38	M	2	8	MD	+		+							
39	M	1	4	MD	+								+	
40	M	7	13	DMD	+	+								+
41	M	2	6	MD	+		+	+						
42	M	0.25	0.25	MD			+		+					
43	M	2	14	DMD	+	+	+	+						
44	M	1	1	MD			+		+					
45	M	1	2	MD	+		+	+						
46	M	1	1	MD			+							
47	M	8	8	MD	+		+		+					
48	M	1	5	MD	+		+							
49	M	12	23	BMD	+		+	+					+	
50	M	0.7	4	DMD	+		+	+			+		+	
51	M	2	10	DMD	+			+					+	

*F* female; *M* male; *DMD* Duchenne muscular dystrophy; *BMD* Becker muscular dystrophy; *MD* muscular dystrophy; + : The patient exhibits the symptom or has the corresponding condition

### Short tandem repeat analysis

Genomic DNA (gDNA) was extracted from the peripheral blood of the patients using the QIAamp DNeasy Blood &Tissue Kit (QIAGEN, Hilde, Germany). The extracted gDNA was amplified by polymerase chain reaction (PCR) using a 2X Goldstar Mix kit (Darui Biotechnology, China). In addition, 20 polymorphic microsatellite loci on chromosomes 13, 18, 21, X, and Y were detected using capillary electrophoresis on an ABI 3500Dx gene sequencer (Applied Biosystems Life Technologies, Singapore). GeneMapper software (version 4.0; Thermo Fisher Scientific) was used to identify relatedness and maternal cell contamination; thus, missed diagnoses and misdiagnoses caused by unrelated relationships or maternal blood contamination were prevented.

### Whole-genome capture of the DMD gene and NGS

The DNA double-stranded capture probe M010 (MyGenostics, Beijing, China) was designed for the whole-genome regions of the DMD gene. The captured libraries were enriched and purified, and then the library concentration was determined by a 7500 Fast Dx Real-Time PCR Instrument, namely a qPCR analyzer (Thermo Fisher Scientific, USA). Paired-end sequencing was performed using the Illumina NextSeq 500/550 medium flux v2 kit (300 cycles; Illumina, California, USA) and the NextSeq 500 platform (Illumina, California, USA), with an average sequencing depth of > 200 × . The sequencing data were compared to the human reference genome (Grch37/ HG19), and base variation was analyzed and annotated using the following databases: BWA (https://www.plob.org/tag/bwa), ANNOVAR (http://annovar.openbioinformatics.org/en/latest/), GATK (http://www.broadinstitute.org/gatk/), gnomAD (http://gnomad.broadinstitute.org/), Esp6500 (http://evs.gs.washington.edu/eVS),1000G (http://browser.1000genomes.org), EXAC (http://exac.broadinstitute.org/), HGMD (https://portal.biobase-international.com/cgi-bin/portal/login.cgi), CLINVAR (https://www.ncbi.nlm.nih.gov/clinvar/) and LOVD (http://www.dmd.nl/nmdb2/home.php). The NCBI Reference Sequence for DMD is NM_004006.3. The pathogenicity classification of the sequence variation was performed according to the standards and guidelines for the interpretation of sequence variants recommended by the American College of Medical Genetics and Genomics and the Association for Molecular Pathology [11].

### Multiplex ligation-dependent probe amplification

The MLPA P034/P035 kit (MRC, Holland) was used to validate exon deletion/duplication of DMD. The DNA amplification products were subjected to capillary electrophoresis with an ABI 3500Dx Genetic Analyzer (Applied Biosystems Life Technologies, Singapore), and the results were analyzed using MLPA Cofalyser.net (MRC, Holland) software.

### Sanger sequencing

Sanger sequencing was used to verify minor variations detected by DMD gene capture and NGS. The sequencing results were analyzed using Chromas software (version 2.4.1; Biosoft).

### Genetic testing of other family members and prenatal diagnosis

gDNA was extracted from the peripheral blood of the other family members, amniotic fluid cells or chorionic villus samples of the fetuses using the QIAamp DNeasy Blood &Tissue Kit (QIAGEN, Hilde, Germany). If the proband had large CNVs in the exon regions of the DMD gene, other family members and fetuses were tested by MLPA. Sanger sequencing was used to detect the variants in the other family members and fetuses for minor variations. After the birth of the fetuses, they received regular physical examinations in the pediatric outpatient department.

## Results

**Fig. 1:**
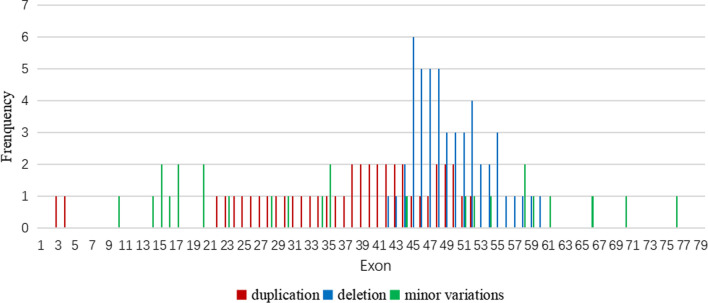
Frequency of each exon variants (deletion, duplication and minor variations) involved in DMD patients. The horizontal coordinate represents the exons 1–79, and the vertical coordinate shows the variant frequency of the exon in this study. Green represents the frequency of small variations (such as single nucleotide variation and < 50 bp fragment insertion/deletion), blue stands for the frequency of exon deletion, and red means the frequency of exon duplication. Exons 45–52 were the most frequently deleted for DMD patients

**Fig. 2:**
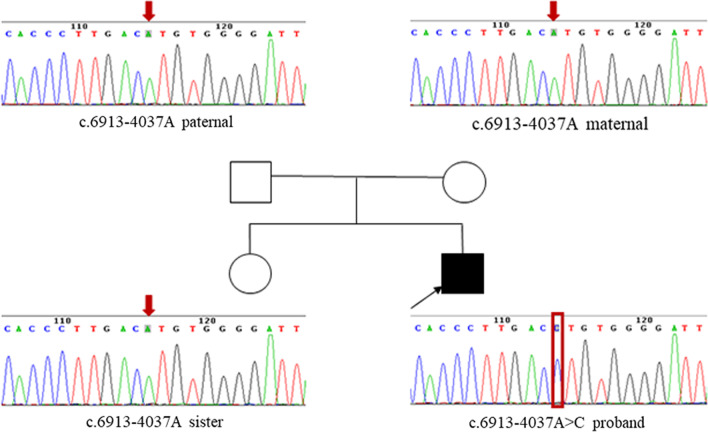
The pedigree of No. 23 patient and the Sanger sequencing results of c.6913-4037A > C

In this study, the DMD gene variants were detected in 49 (96.1% [49/51]) families, and a total of 45 variant types were identified, wherein large fragment deletions, duplications, and minor variations accounted for 25.5% (13/51), 7.8% (4/51), and 62.7% (32/51), respectively (Table 2). Exon deletions were distributed in the regions of exons 42–60, and the frequency of deletions was highest in exons 45–52, accounting for 68.0% (34/50) of deletions. Single exon deletion was found in six cases, and exon 45 deletion accounted for 23.1% (3/13) of exon deletions. There were no obvious variant hotspots in the entire regions for exon duplications or minor variations (Fig. 1). Thirty-two minor variations were detected, including nineteen nonsense variants (59.4% [19/32]), seven frameshift variants (21.9% [7/32]), and six splicing substitutions (18.8% [6/32]). Among the 49 patients with identified pathogenic variants, 43 patients had variants located in exon regions, five patients had variants located in exon flanking regions, and one patient had a variant in the deep intronic region (Patient No. 23). The variant (c.6913–4037 A > C) of No. 23 was not recorded at this locus in the peripheral blood DNA of the parents and his elder sister (Fig. 2).

**Table 2:** 49 variants detected in dystrophinopathy cases

NO	Exon/intron	Nucleotide change	Amino acid change	Het/hemi	Inheritance	Prenatal diagnosis		References
						Genetic testing	Pregnancy outcome	
1	Exo 70	c.10171C > T	p.R3391*	Hemi	Mother	Het	No abnormal phenotype	Barbieri et al. [37] Eur J Hum Genet 4,183
2	Exo 10	c.1075G > T	p.E359*	Hemi	De novo			Tong et al. [21] Front Neurol 11:e572006
3	Exo 76	c.10916_10917del	p.S3639Yfs*3	Hemi	Mother			Novel
4	Int 11	c.1332-9A > G	splicing	Hemi	Mother	Normal	No abnormal phenotype	Flanigan et al. [38] Am J Hum Genet 72,931
5	Exo 14	c.1684C > T	p.Q562*	Hemi	Mother			Takeshima et al. [17] J Hum Genet 55,379
6	Exo 15	c.1790 T > A	p.L597*	Hemi	Mother			Novel
7	Exo 15	c.1793C > G	p.S598*	Hemi	De novo			Flanigan et al. [39] Hum Mutat 30,1657
8	Exo 16	c.1842del	p.K614Nfs*18	Hemi	Mother			Novel
9	Int 3	c.186 + 2 T > C	splicing	Hemi	Mother	Hemi	Termination	Flanigan et al. [39] Hum Mutat 30,1657
10	Int 16	c.1992 + 1G > C	splicing	Hemi	Mother			Neri et al. [40] Front Genet 11,131
11	Exo 17	c.2111del	p.P704Hfs*25	Hemi	Mother			Santos et al. [19] J Hum Genet 59,454
12	Exo 17	c.2128A > T	p.K710*	Hemi	Mother			Mendell et al. [41] Neurology 57,645
13	Exo 20	c.2461G > T	p.E821*	Hemi	Mother			Clinvar
14	Exo 20	c.2484 T > A	p.Y828*	Hemi	Mother			Guo et al. [22] J Hum Genet 60,435
15	Exo 23	c.3151C > T	p.R1051*	Hemi	Mother			Bennett et al. [42] BMC Genet 2,17
16	Int 25	c.3432 + 1G > T	splicing	Hemi	Mother			Hofstra et al. [43] Hum Mutat 23,57
17	Exo 28	c.3856G > T	p.E1286*	Hemi	Mother	Het	No abnormal phenotype	Clinvar
18	Exo 30	c.4108C > T	p.Q1370*	Hemi	Mother			Tuffery-Giraud et al. [44] Neuromuscul Disord 14,650
19	Exo 34	c.4841del	p.G1614Efs*15	Hemi	Mother			Tuffery-Giraud et al. [44] Neuromuscul Disord 14,650
20	Exo 35	c.5015del	p.N1672Ifs*49	Hemi	Mother			Novel
21	Exo 35	c.5791_5792insCA	p.R1931Tfs*53	Hemi	De novo			Novel
22	Exo 44	c.6292C > T	p.R2098*	Hemi	Mother	Normal	No abnormal phenotype	Roberts et al. [45] Hum Mutat 4,1
23	Int 47	c.6913-4037A > C	Splicing	HEMI	De novo			Gurvich et al. [46] Ann Neurol 63,81
24	Exo 51	c.7339C > T	p.Q2447*	Hemi	De novo			Dolinsky et al. [47] Neuromuscul Disord 12,845
25	Exo 52	c.7657C > T	p.R2553*	Hemi	Mother			Lesca et al. [48] Rev Neurol 159,775
26	Int 52	c.7661-1G > A	Splicing	Hemi	Mother			Ma [34] Orphanet J Rare Dis 13,109
27	Exo 54	c.7885_7886del	p.D2629Pfs*13	Hemi	Mother			Xu et al. [49] J Clin Lab Anal 32:e22445
28,29	Exo 58	c.8608C > T	p.R2870*	Hemi	Mother			Mendell et al. [41] Neurology 57,645
30	Exo 59	c.8713C > T	p.R2905*	Hemi	Mother			Prior et al. [50] Am J Hum Genet 57,22
31	Exo 61	c.9148C > T	p.Q3050*	Hemi	De novo			Tuffery-Giraud et al. [44] Neuromuscul Disord 14,650
32	Exo 66	c.9568C > T	p.R3190*	Hemi	Mother	Normal	No abnormal phenotype	Tuffery et al. [51] Hum Genet 102,334
33	Exo 38–50	Duplication		Het	Uncertain			Novel
34,42	Exo 44	Deletion		Hemi	Mother			Chen et al. [52] PLoS One 9:e108038
35,38,45	Exo 45	Deletion		Hemi	Uncertain			Chen et al. [52] PLoS One 9:e108038
36	Exo 55–60	Deletion		Hemi	Uncertain			Chen et al. [53] Electrophoresis 34,2503
37	Exo 52	Deletion		Hemi	Mother			Hrdlicka et al. [54] Folia Biol (Praha) 47,81
39	Exo 22–44	Duplication		Hemi	Mother	Normal	No abnormal phenotype	Zimowski et al. [55] Med Wieku Rozwoj 13,140
40	Exo 45–52	Deletion		Hemi	Mother			Moizard et al. [56] Am J Med Genet 80,32
41	Exo 46–55	Deletion		Hemi	Mother			Moizard et al. [56] Am J Med Genet 80,32
43	Exo 48–52	Duplication		Hemi	Uncertain			Moizard et al. [56] Am J Med Genet 80,32
44	Exo 46–48	Deletion		Hemi	Uncertain			Sinha et al. [57] Clin Genet 50,327
46	Exo 3–4	Duplication		Hemi	Mother			Hu et al. [58] Am J Hum Genet 46,682
47	Exo 42–43	Deletion		Hemi	Mother			Janssen et al. [59] Neurogenetics 6,29
48	Exo 45–55	Deletion		Hemi	Uncertain			Lalic et al. [60] Eur J Hum Genet 13,1231
49	Exo 45–48	Deletion		Hemi	Mother	Normal	No abnormal phenotype	Beggs et al. [61] Am J Hum Genet 49,54

*Exo* exon; *Int* intron; *Het* heterozygous; *Hemi* hemizygous

**Fig. 3:**
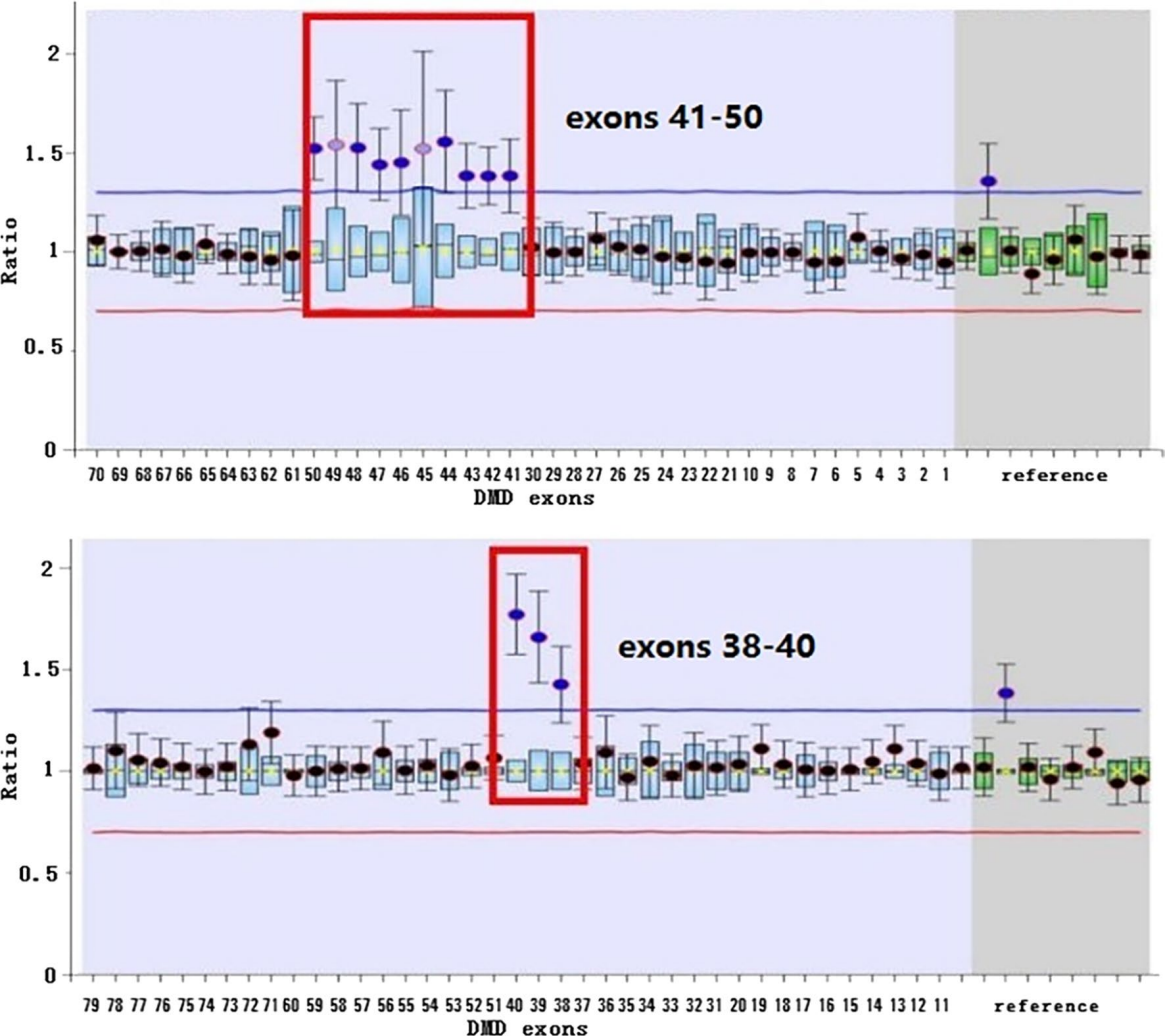
The results of MLPA for exons 38–50 heterozygous duplication of No.33 female patient

Among the 49 DMD gene variants, 43 were known pathogenic variants and 6 were novel variants. A female patient (No.33) with detectable duplication of exons 38–50 was hospitalized for increased CK and CK-MB and left ventricular enlargement, and the results of MLPA are shown in Fig. 3. The other five novel minor variations included one nonsense variant (c.1790 T > A) and four frameshift variants (c.10916_10917del, c.1842del, c.5015del, and c.5791_5792insCA). The six novel variants were classified as pathogenic variants (PVS1 + PM2 + PP4). Furthermore, none of these six variants had previously been reported in public databases.

Mothers of 41 of the patients underwent DMD gene testing, and 35 patients (85.4% [35/41]) were found to be carriers of gene variants such as nine exon deletions/duplications and 26 minor variations. The mothers of six DMD patients (14.6% [6/41]) did not carry the same pathogenic variants as their children, and all of them had no family history; however, whether the mothers had germline mosaicism was uncertain. In this study, eight carriers (including seven mothers and one sister) completed a prenatal diagnosis for the DMD gene in our hospital. Five fetuses (62.5% [5/8]) did not carry the corresponding variant and two fetuses (25.0% [2/8]) had heterozygous variants. While all the babies born were healthy, one fetus (12.5% [1/8]) was subject to a hemizygous variant, and the pregnancy was terminated (Table 2).

## Discussion

The diagnoses of DMD/BMD can be divided into clinical, pathological, and genetic diagnosis. The clinical criteria for diagnoses include muscle strength, electromyography, and biochemistry. The relevant testing items are easy to implement, but the motor development of infants with DMD/BMD is immature and lacks typical clinical manifestations, such as muscle weakness and atrophy. Some patients only showed elevated serum CK/CK-MB levels, which may be due to the young age at first diagnosis, lack of movement disorders, and insignificant DMD/BMD-related symptoms. In this study, nine patients showed no evidence of movement disorders, only elevated CK and CK-MB levels. Three of these patients had been misdiagnosed with “myocarditis” in other hospitals. The family members of the 42 patients with motor retardation could detect the abnormalities and therefore, the patients were diagnosed promptly. However, for infants only with “CK/CK-MB increase”, the diagnosis can be easily missed. Therefore, attention needs to be paid to clinical features, and genetic testing should be performed as soon as possible to make a clear diagnosis. Female DMD carriers are usually asymptomatic, but some may still experience symptoms due to X-chromosome inactivation [12]. In female DMD carriers, the prevalence of elevated CK was approximately 53%, skeletal muscle injury was approximately 2.5–19%, and dilated cardiomyopathy was approximately 7.3–16.7% [13]. In this study, the case (No.33) was a female carrier with heterozygous duplications of exons 38–50, clinically characterized by an increase in CK and CK-MB and left ventricular enlargement.

Pathological diagnosis aims to detect necrosis of muscle cells, fiber size, and the degree of fibrosis through a muscle biopsy and immunohistochemical staining. A Muscle biopsy is an invasive test that is hardly accepted by family members and children. In addition, the efficacy of a muscle biopsy is limited by the degree of disease progression, sampling position, and laboratory technology, therefore test results are generally inaccurate [14]. Consequently, with the rapid development of molecular biology techniques, gene detection has become the primary method for the diagnosis of DMD/BMD. Technologies such as MLPA, Sanger sequencing, mPCR, and ES are commonly used. MLPA can detect large deletions/duplications in the exon regions of the DMD gene, but not minor variants. The MLPA test result is negative, which can prolong the testing time and even lead to a missed diagnosis. In our study, patients who had previously tested negative for MLPA took 1 to 18 years to receive a definitive genetic diagnosis of the DMD gene, and some patients even died before the test results were available. Sanger sequencing is generally only used to validate known variant sites. Exon sequencing technology is limited by its relatively high economic cost and its failure to comprehensively cover the entire genetic region of DMD. In other studies, capture-based NGS detection can only detect exon and flanking intron regions (< 30 bp) due to the incomplete capture region of the probe [15]. For example, for case No. 23, the pathogenic variant was located in intron 47 (c.6913–4037), which is a variant in the deep-intronic region. Therefore, if ES and the other capture-based NGS had been performed, the variant might not have been detected. According to De Palma FDE et al. [16], the authors compared capture-based NGS with traditional methods (MLPA/mPCR/Sanger sequencing) and concluded that capture-based NGS overcomes the limitations of MLPA and Sanger sequences. The capture-based NGS is easy to apply in clinical practice and should be the main strategy for the diagnosis of DMD. In this study, whole-genome capture of the DMD gene and NGS can detect both large fragment deletions/duplications, and minor variants, especially for deep intronic regions, so whole-genome capture of the DMD gene and NGS is recommended as the main strategy for diagnosis of DMD, which not only improves the detection rate but also shortens the time to diagnosis.

Worldwide, DMD variants are mainly exon deletions or duplications, accounting for 70–83.1%, with minor variations accounting for only 16.9–30% [17–19]. In studies in China, exon deletions or duplications accounted for 65.5–79.2%, with minor variations accounting for only 14.4–23.3% [20–22]. Our results differ from other studies, exon deletions or duplications and minor variations of 34.7% and 65.3%, respectively. MLPA is relatively fast and low cost, which is a reasonable choice for designing a testing algorithm for DMD/BMD patients when new technologies are not available. Southwest China is economically backward and some patients prefer cheaper techniques (e.g., MLPA), while most genetic laboratories can only offer MLPA testing due to limitations in technology, equipment, and talent shortage. The genetic counsellor may consider financial or other issues first and test the patient for MLPA first. If no abnormality was detected by MLPA, then the patient underwent whole-genome capture of the DMD gene and NGS. Therefore, compared with other studies, relatively few cases had CNVs in the DMD gene. As found in this study, the DMD exon deletion was commonly located in the exon 45–52 region, namely the central structural region, and these results were similar to those obtained in previous studies [20, 23]. In addition, the distribution of minor variations in the DMD gene region did not have hotspots; these results were also consistent with previous studies [21]. No definite pathogenic or possible pathogenic variations were detected in two patients (nos. 50/51) with DMD. Their clinical manifestations were consistent with the DMD phenotype, which was confirmed by muscle biopsy. This may be caused by complex variations in the DMD gene, such as rearrangements, non-DMD gene sequence insertions, translocation variants, and inversions. This can alter the normal sequence of the DMD gene and prevent the production of full transcripts, resulting in truncated dystrophin [24].

Patients with DMD/BMD are subject to high mortality and poor prognosis, and there is currently no effective way to cure DMD/BMD. Additionally, DMD patients cause huge economic and psychological burdens to families and society [25]. At present, DMD/BMD is still mainly treated with neurological-based multidisciplinary comprehensive treatment. However, gene therapy is currently a research hotspot. In approximately 10–15% of DMD/BMD patients with nonsense variants, early stop codon readthrough can be induced to continue the translation of dystrophin [26, 27]. The drug PTC124 (3-5-(2-fluorophenyl)-1,2,4oxadiazol-3-yl-benzoic acid; C_15_H_9_FN_2_O_3_) is currently being evaluated in clinical trials for the treatment of nonsense variants [28]. In addition, antisense oligonucleotide-mediated exon skipping produces efficient target exon skipping by regulating the splicing of mRNA precursors. Thus, the transcription reading frame is restored while a shortened but partially functional anti-atrophy protein is produced [29]. In other studies, adeno-associated viral vectors have been used to deliver specific gRNA/Cas9 into MDX skeletal and cardiac muscles, which can remove one or more exons from genomic DNA so that truncated, but partially functional anti-muscular dystrophy proteins can be produced [30–32]. Nevertheless, the above-mentioned gene therapy plans for DMD/BMD still need to clarify the location and type of gene variant. Therefore, prenatal diagnosis is the only effective way to reduce the number of children with DMD/BMD, and a clear genetic diagnosis is the premise of prenatal diagnosis.

Our study showed that the de novo variant rate of DMD was 14.6% (6/41), and 85.4% (35/41) of the DMD gene variants were inherited. The probability of inheritance is related to the type of DMD gene variant. The total inheritance probability (60.0–86.5%) was similar to that in other studies [18, 33–35]. If the DMD variant in a patient was a de novo variant, the possibility of germline mosaicism cannot be ruled out. As shown in some studies, for women who have previously given birth to children with DMD/BMD, if DMD genetic variation consistent with the children is not detected in their peripheral blood, the risk of having another child with DMD/BMD is due to different types of DMD genetic variation, with the total genetic risk being approximately 4.3% [36]. For families of DMD patients with unknown variants, if female members wish to become pregnant, third-generation sequencing may help to detect the DMD gene variant sites of patients or the sex chromosome of the fetus could be determined for gender selection. For families with children with DMD/BMD, healthy carriers in the family should be identified. Appropriate genetic counseling should be provided to high-risk couples to ensure access to pre-implantation and/or prenatal diagnosis. In this study, eight patients (15.7% [8/51]) had a family history. In the family of Patient No. 40, the mother's first child died in early childhood, and the second and third children had DMD. However, the family members did not have enough knowledge of the disease and did not receive genetic counseling or prenatal diagnosis during pregnancy, which is why another DMD/BMD-affected child was born. Therefore, child health care and pre-pregnancy consultation should be strengthened, and timely prenatal diagnosis should be used when necessary to avoid the birth of children with this genetic disease.

## Conclusions

In this study, we analyzed the DMD gene using whole-genome capture of the DMD gene and NGS from 51 DMD/BMD patients in southwestern China. Pathogenic or likely pathogenic variants were detected in 49 patients (96.1% [49/51]), of which DMD variants in 6 patients (12.2% [6/48]) had not been previously reported. This study not only confirmed the value of whole-genome capture of the DMD gene and NGS but also expanded the gene variant spectrum of DMD, which may improve genetic counseling and prenatal diagnosis.

## Data Availability

All data generated or analysed in this study are included in this published article and Table 2. The raw datasets used and analysed during the current study are not deposited in publicly available repositories because of considerations about the security of human genetic resources. For other details of the availability of data and material, please refer to the methods section of the article and tables. The sequencing dataset can be obtained from the corresponding author on reasonable request.

## Abbreviations

DMDL: Duchenne muscular dystrophy
BMD: Becker muscular dystrophy
MD: Muscular dystrophy
MLPA: Multiplex ligation-dependent probe amplification
ES: Exome sequencing
NGS: Next-generation sequencing
CNV: Copy number variation
CK: Creatine kinase
CK-MB: Creatine kinase isoenzyme MB
PCR: Polymerase chain reaction

## References

[1] Dunnen JT, Grootscholten PM, Dauwerse JG, Walker AP, Monaco AP, Butler R (1992). Reconstruction of the 2.4 Mb human DMD-gene by homologous YAC recombination. Hum Mol Genet.

[2] Bushby K, Finkel R, Birnkrant DJ, Case LE, Clemens PR, Cripe L (2010). DMD Care Considerations Working Group. Diagnosis and management of Duchenne muscular dystrophy, part 1: diagnosis, and pharmacological and psychosocial management. Lancet Neurol.

[3] Theadom A, Rodrigues M, Roxburgh R, Balalla S, Higgins C, Bhattacharjee R (2014). Prevalence of muscular dystrophies: a systematic literature review. Neuroepidemiology.

[4] Chung J, Smith AL, Hughes SC, Niizawa G, Abdel-Hamid HZ, Naylor EW (2016). Twenty-year follow-up of newborn screening for patients with muscular dystrophy. Muscle Nerve.

[5] Danieli GA, Mostacciuolo ML, Bonfante A, Angelini C (1977). Duchenne muscular dystrophy. A population study. Hum Genet.

[6] Danieli GA, Mostacciuolo ML, Pilotto G, Angelini C, Bonfante A (1980). Duchenne muscular dystrophy: data from family studies. Hum Genet.

[7] König K, Pechmann A, Thiele S, Walter MC, Schorling D, Tassoni A (2019). De-duplicating patient records from three independent data sources reveals the incidence of rare neuromuscular disorders in Germany. Orphanet J Rare Dis.

[8] Wein N, Alfano L, Flanigan KM (2015). Genetics and emerging treatments for Duchenne and Becker muscular dystrophy. Pediatr Clin North Am.

[9] da Silva TD, Massetti T, Crocetta TB, de Mello Monteiro CB, Carll A, Vanderlei LCM (2018). Heart rate variability and cardiopulmonary dysfunction in patients with Duchenne muscular dystrophy: a systematic review. Pediatr Cardiol.

[10] Muntoni F, Torelli S, Ferlini A (2003). Dystrophin and mutations: one gene, several proteins, multiple phenotypes. Lancet Neurol.

[11] Richards S, Aziz N, Bale S, Bick D, Das S, Gastier-Foster J (2015). Standards and guidelines for the interpretation of sequence variants: a joint consensus recommendation of the American College of Medical Genetics and Genomics and the Association for Molecular Pathology. Genet Med.

[12] Brioschi S, Gualandi F, Scotton C, Armaroli A, Bovolenta M, Falzarano MS (2012). Genetic characterization in symptomatic female DMD carriers: lack of relationship between X-inactivation, transcriptional DMD allele balancing and phenotype. BMC Med Genet.

[13] Ishizaki M, Kobayashi M, Adachi K, Matsumura T, Kimura E (2018). Female dystrophinopathy: review of current literature. Neuromuscul Disord.

[14] Barthelemy F, Woods JD, Nieves-Rodriguez S, Douine ED, Wang R, Wanagat J (2020). A well-tolerated core needle muscle biopsy process suitable for children and adults. Muscle Nerve.

[15] Okubo M, Minami N, Goto K, Goto Y, Noguchi S, Mitsuhashi S (2016). Genetic diagnosis of Duchenne/Becker muscular dystrophy using next-generation sequencing: validation analysis of DMD mutations. J Hum Genet.

[16] De Palma FDE, Nunziato M, D'Argenio V, Savarese M, Esposito G, Salvatore F (2021). Comprehensive molecular analysis of DMD gene increases the diagnostic value of dystrophinopathies: a pilot study in a Southern Italy cohort of patients. Diagnostics (Basel, Switzerland).

[17] Takeshima Y, Yagi M, Okizuka Y, Awano H, Zhang Z, Yamauchi Y (2010). Mutation spectrum of the dystrophin gene in 442 Duchenne/Becker muscular dystrophy cases from one Japanese referral center. J Hum Genet.

[18] Cho A, Seong MW, Lim BC, Lee HJ, Byeon JH, Kim SS (2017). Consecutive analysis of mutation spectrum in the dystrophin gene of 507 Korean boys with Duchenne/Becker muscular dystrophy in a single center. Muscle Nerve.

[19] Santos R, Gonçalves A, Oliveira J, Vieira E, Vieira JP, Evangelista T (2014). New variants, challenges and pitfalls in DMD genotyping: implications in diagnosis, prognosis and therapy. J Hum Genet.

[20] Li X, Zhao L, Zhou S, Hu C, Shi Y, Shi W (2015). A comprehensive database of Duchenne and Becker muscular dystrophy patients (0–18 years old) in East China. Orphanet J Rare Dis.

[21] Tong YR, Geng C, Guan YZ, Zhao YH, Ren HT, Yao FX (2020). A comprehensive analysis of 2013 dystrophinopathies in China: a report from National Rare Disease Center. Front Neurol.

[22] Guo R, Zhu G, Zhu H, Ma R, Peng Y, Liang D (2015). DMD mutation spectrum analysis in 613 Chinese patients with dystrophinopathy. J Hum Genet.

[23] Beggs AH, Koenig M, Boyce FM, Kunkel LM (1990). Detection of 98% of DMD/BMD gene deletions by polymerase chain reaction. Hum Genet.

[24] Trippe H, Wieczorek S, Kötting J, Kress W, Schara U (2014). Xp21/A translocation: a rarely considered genetic cause for manifesting carriers of duchenne muscular dystrophy. Neuropediatrics.

[25] Labisa P, Andreozzi V, Mota M, Monteiro S, Alves R, Almeida J (2021). Cost of Illness in patients with Duchenne muscular dystrophy in Portugal: the COIDUCH study. Pharmacoecon Open.

[26] Mah JK, Selby K, Campbell C, Nadeau A, Tarnopolsky M, McCormick A (2011). A population-based study of dystrophin mutations in Canada. Can J Neurol Sci.

[27] Bladen CL, Rafferty K, Straub V, Monges S, Moresco A, Dawkins H (2013). The TREAT-NMD Duchenne muscular dystrophy registries: conception, design, and utilization by industry and academia. Hum Mutat.

[28] Welch EM, Barton ER, Zhuo J, Tomizawa Y, Friesen WJ, Trifillis P (2007). PTC124 targets genetic disorders caused by nonsense mutations. Nature.

[29] Echigoya Y, Yokota T (2014). Skipping multiple exons of dystrophin transcripts using cocktail antisense oligonucleotides. Nucleic Acid Ther.

[30] Lim KRQ, Yoon C, Yokota T (2018). Applications of CRISPR/Cas9 for the treatment of Duchenne muscular dystrophy. J Pers Med.

[31] Zhang Y, Li H, Min YL, Sanchez-Ortiz E, Huang J, Mireault AA (2020). Enhanced CRISPR-Cas9 correction of Duchenne muscular dystrophy in mice by a self-complementary AAV delivery system. Sci Adv.

[32] Min YL, Li H, Rodriguez-Caycedo C, Mireault AA, Huang J, Shelton JM (2019). CRISPR-Cas9 corrects Duchenne muscular dystrophy exon 44 deletion mutations in mice and human cells. Sci Adv.

[33] Zhang J, Ma D, Liu G, Wang Y, Liu A, Li L (2019). Genetic analysis of 62 Chinese families with Duchenne muscular dystrophy and strategies of prenatal diagnosis in a single center. BMC Med Genet.

[34] Ma P, Zhang S, Zhang H, Fang S, Dong Y, Zhang Y (2018). Comprehensive genetic characteristics of dystrophinopathies in China. Orphanet J Rare Dis.

[35] Roucher Boulez F, Menassa R, Streichenberger N, Manel V, Mallet-Motak D, Morel Y (2015). A splicing mutation in the DMD gene detected by next-generation sequencing and confirmed by mRNA and protein analysis. Clin Chim Acta.

[36] Helderman-van den Enden AT, de Jong R, den Dunnen JT, Houwing-Duistermaat JJ, Kneppers AL, Ginjaar HB (2009). Recurrence risk due to germ line mosaicism: Duchenne and Becker muscular dystrophy. Clin Genet.

[37] Barbieri AM, Soriani N, Ferlini A, Michelato A, Ferrari M, Carrera P (1996). Seven novel additional small mutations and a new alternative splicing in the human dystrophin gene detected by heteroduplex analysis and restricted RT-PCR heteroduplex analysis of illegitimate transcripts. Eur J Hum Genet.

[38] Flanigan KM, von Niederhausern A, Dunn DM, Alder J, Mendell JR, Weiss RB (2003). Rapid direct sequence analysis of the dystrophin gene. Am J Hum Genet.

[39] Flanigan KM, Dunn DM, von Niederhausern A, Soltanzadeh P, Gappmaier E, Howard MT (2009). Mutational spectrum of DMD mutations in dystrophinopathy patients: application of modern diagnostic techniques to a large cohort. Hum Mutat.

[40] Neri M, Rossi R, Trabanelli C, Mauro A, Selvatici R, Falzarano MS (2020). The genetic landscape of dystrophin mutations in Italy: a nationwide study. Front Genet..

[41] Mendell JR, Buzin CH, Feng J, Yan J, Serrano C, Sangani DS (2001). Diagnosis of Duchenne dystrophy by enhanced detection of small mutations. Neurology..

[42] Bennett RR, den Dunnen J, O'Brien KF, Darras BT, Kunkel LM (2001). Detection of mutations in the dystrophin gene via automated DHPLC screening and direct sequencing. BMC Genet..

[43] Hofstra RM, Mulder IM, Vossen R, de Koning-Gans PA, Kraak M, Ginjaar IB (2004). DGGE-based whole-gene mutation scanning of the dystrophin gene in Duchenne and Becker muscular dystrophy patients. Hum Mutat..

[44] Tuffery-Giraud S, Saquet C, Chambert S, Echenne B, Marie Cuisset J, Rivier F (2004). The role of muscle biopsy in analysis of the dystrophin gene in Duchenne muscular dystrophy: experience of a national referral centre. Neuromuscul Disord..

[45] Roberts RG, Gardner RJ, Bobrow M (1994). Searching for the 1 in 2,400,000: a review of dystrophin gene point mutations. Hum Mutat..

[46] Gurvich OL, Tuohy TM, Howard MT, Finkel RS, Medne L, Anderson CB (2008). DMD pseudoexon mutations: splicing efficiency, phenotype, and potential therapy. Ann Neurol..

[47] Dolinsky LC, de Moura-Neto RS, Falcão-Conceição DN (2002). DGGE analysis as a tool to identify point mutations, de novo mutations and carriers of the dystrophin gene. Neuromuscul Disord..

[48] Lesca G, Demarquay G, Llense S, Streichenberger N, Petiot P, Michel-Calemard L (2003). Les conductrices symptomatiques pour les dystrophinopathies avec biais d'inactivation du chromosome X [Symptomatic carriers of dystrophinopathy with chromosome X inactivation bias]. Rev Neurol (Paris)..

[49] Xu Y, Li Y, Song T, Guo F, Zheng J, Xu H (2018). A retrospective analysis of 237 Chinese families with Duchenne muscular dystrophy history and strategies of prenatal diagnosis. J Clin Lab Anal..

[50] Prior TW, Bartolo C, Pearl DK, Papp AC, Snyder PJ, Sedra MS (1995). Spectrum of small mutations in the dystrophin coding region. Am J Hum Genet..

[51] Tuffery S, Chambert S, Bareil C, Sarda P, Coubes C, Echenne B (1998). Mutation analysis of the dystrophin gene in Southern French DMD or BMD families: from Southern blot to protein truncation test. Hum Genet..

[52] Chen C, Ma H, Zhang F, Chen L, Xing X, Wang S (2014). Screening of Duchenne muscular dystrophy (DMD) mutations and investigating its mutational mechanism in Chinese patients. PLoS One..

[53] Chen CA, Chang MY, Chang TM, Jong YJ, Wu SM (2013). Capillary electrophoresis for analysis of deletion and duplication in exon 44–55 of Duchenne muscular dystrophy gene. Electrophoresis..

[54] Hrdlicka I, Zadina J, Krejcí R, Srbová A, Kucerová M (2001). Patterns of deletions and the distribution of breakpoints in the dystrophin gene in Czech patients with Duchenne and Becker muscular dystrophy (statistical comparison with results from several other countries). Folia Biol (Praha)..

[55] Zimowski JG, Holding M, Fidziańska E, Fidziańska A, Ryniewicz B, Dobosz I (2009). Wykrywanie rzadkich mutacji w genie dystrofiny [Detection of rare mutations in the dystrophin gene]. Med Wieku Rozwoj..

[56] Moizard MP, Billard C, Toutain A, Berret F, Marmin N, Moraine C (1998). Are Dp71 and Dp140 brain dystrophin isoforms related to cognitive impairment in Duchenne muscular dystrophy?. Am J Med Genet..

[57] Sinha S, Mishra S, Singh V, Mittal RD, Mittal B (1996). High frequency of new mutations in North Indian Duchenne/Becker muscular dystrophy patients. Clin Genet..

[58] Hu XY, Ray PN, Murphy EG, Thompson MW, Worton RG (1990). Duplicational mutation at the Duchenne muscular dystrophy locus: its frequency, distribution, origin, and phenotypegenotype correlation. Am J Hum Genet..

[59] Janssen B, Hartmann C, Scholz V, Jauch A, Zschocke J (2005). MLPA analysis for the detection of deletions, duplications and complex rearrangements in the dystrophin gene: potential and pitfalls. Neurogenetics..

[60] Lalic T, Vossen RH, Coffa J, Schouten JP, Guc-Scekic M, Radivojevic D, Djurisic M, Breuning MH, White SJ, den Dunnen JT (2005). Deletion and duplication screening in the DMD gene using MLPA. Eur J Hum Genet..

[61] Beggs AH, Hoffman EP, Snyder JR, Arahata K, Specht L, Shapiro F (1991). Exploring the molecular basis for variability among patients with Becker muscular dystrophy: dystrophin gene and protein studies. Am J Hum Genet..

